# Green Tea Intake and Risks for Dementia, Alzheimer’s Disease, Mild Cognitive Impairment, and Cognitive Impairment: A Systematic Review

**DOI:** 10.3390/nu11051165

**Published:** 2019-05-24

**Authors:** Saki Kakutani, Hiroshi Watanabe, Norihito Murayama

**Affiliations:** Research Institute, Suntory Global Innovation Center Limited, Suntory World Research Center, 8-1-1 Seikadai, Seika-cho, Souraku-gun, Kyoto 619-0284, Japan; H_Watanabe@suntory.co.jp (H.W.); Norihito_Murayama@suntory.co.jp (N.M.)

**Keywords:** green tea intake, beverage, dementia, Alzheimer’s disease, mild cognitive impairment, cognitive impairment, systematic review, observational studies, free-living populations, elderly

## Abstract

Dementia has become a major issue that requires urgent measures. The prevention of dementia may be influenced by dietary factors. We focused on green tea and performed a systematic review of observational studies that examined the association between green tea intake and dementia, Alzheimer’s disease, mild cognitive impairment, or cognitive impairment. We searched for articles registered up to 23 August 2018, in the PubMed database and then for references of original articles or reviews that examined tea and cognition. Subsequently, the extracted articles were examined regarding whether they included original data assessing an association of green tea intake and dementia, Alzheimer’s disease, mild cognitive impairment, or cognitive impairment. Finally, we included three cohort studies and five cross-sectional studies. One cohort study and three cross-sectional studies supported the positive effects of green tea intake. One cohort study and one cross-sectional study reported partial positive effects. The remaining one cohort study and one cross-sectional study showed no significant association of green tea intake. These results seem to support the hypothesis that green tea intake might reduce the risk for dementia, Alzheimer’s disease, mild cognitive impairment, or cognitive impairment. Further results from well-designed and well-conducted cohort studies are required to derive robust evidence.

## 1. Introduction

Dementia has become a major social issue that requires urgent measures to both address and prevent its occurrence. Dementia afflicts around 50 million people worldwide, with 10 million new cases annually [1]. The number of people with dementia is projected to reach 82 million in 2030 and 152 million in 2050 [1]. This rapid increase is due to the aging population, as age is the strongest known risk factor for dementia. Dementia has significant social and economic implications in terms of the costs of direct medical and social care, and of informal care. In 2015, the total global societal cost of dementia was estimated to be $818 billion USD, equivalent to 1.1% of global gross domestic product [1]. Moreover, dementia can cause physical, emotional, and economic pressures that bring on great stress to families of affected people and their carers.

Dietary factors may play a role in the prevention of dementia, among which beverages are considered useful because their intake does not drastically affect other dietary habits and is more acceptable. Tea is one of the most common beverages in the world. The intake of tea, which contains caffeine and tea polyphenols, may be important. High caffeine intake may reduce the risk for dementia [2,3,4]. Particularly in Alzheimer’s disease (AD), the green tea catechin polyphenols have been reported to have neuroprotective effects such as anti-oxidative stress, anti-inflammation, inhibition of amyloid-beta aggregation, and anti-apoptosis [5,6]. A systematic review by Mancini et al., reported beneficial effects of green tea on cognition [7]. Therefore, because green tea is associated with neurodegenerative disease and cognitive functions, we identified the relationship between green tea and AD, mild cognitive impairment (MCI), and cognitive impairment by a systematic review.

We performed a systematic review of observational studies that examined the association between green tea intake and dementia, AD, MCI, or cognitive impairment. We selected dementia, AD, MCI, and cognitive impairment as the outcome because all of them conceptually include cognitive dysfunction that is greater than expected for age and education level without other psychiatric disorders; it should be noted that cognitive impairment is difficult to distinguish from cognitive dysfunction caused by aging, heredity, autism, and intelligence disability.

## 2. Materials and Methods 

### 2.1. Search Strategy

Inclusion criteria were (1) English-language articles, (2) articles published after 1966, (3) articles on free-living populations, and (4) articles reporting original data on the relationship between green tea intake and the risk for dementia, AD, MCI, or cognitive impairment. We included cohort, case-cohort, nested case-control, case-control, and cross-sectional studies.

The article search was conducted using two steps. The first step included a comprehensive listing of articles that examined an association between intake of all kinds of tea and cognitive function or a change of cognitive function, in addition to dementia, AD, MCI, or cognitive impairment. In the second step, we extracted articles from Step 1 that examined intake of only green tea on dementia, AD, MCI, or cognitive impairment.

### 2.2. Study Selection

The PubMed database [8] was searched for observational studies investigating the relationship between tea intake and risk for dementia, AD, MCI, or cognitive impairment up to 23 August 2018. We designed the PubMed search formula as follows: key words for humans AND study designs AND exposure AND relevant outcomes (Table 1).

**Figure 1:**
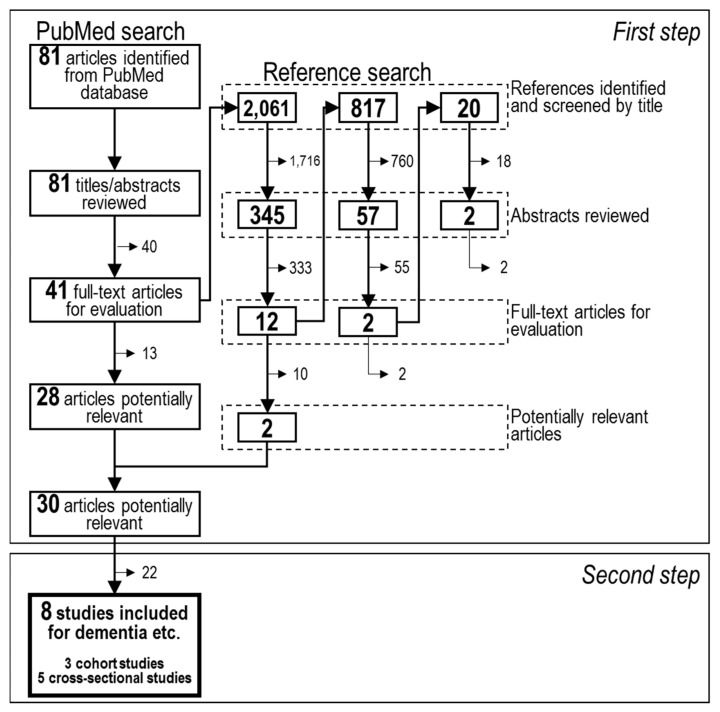
Flow diagram for literature search and study selection.

Figure 1 shows the study selection process. The PubMed search yielded 81 articles. First, we excluded articles in which titles and abstracts met the following criteria: (1) non-English publications, (2) non-human studies, (3) intervention studies, (4) studies that did not examine tea intake as exposure, (5) studies that examined tea intake in combination with other factors such as genotypes of metabolic enzymes or other dietary factors, (6) studies that did not examine dementia, AD, MCI, or cognitive impairment, or (7) studies that were published prior to 1965. Next, each full-text article was reviewed against the following inclusion criteria: (1) studies with eligible design and (2) studies reporting original data of tea intake and risk for dementia, AD, MCI, or cognitive impairment. Twenty-eight eligible articles were obtained from the initial PubMed search.

To obtain additional potentially relevant articles, we assessed the citations of the full-text articles from our PubMed search. Citations of studies that (1) were non-English publications, (2) were non-human studies, (3) were intervention studies, (4) did not examine tea, beverages, diet, food, nutrients, antioxidant, or lifestyles, (5) did not examine dementia, AD, amyloid-beta coagulation, cognition, neurodegeneration, neuroprotection, neural function, memory, psychiatric disorders, or brain aging, and (6) were published prior to 1965 could be immediately excluded. A shortened list of articles from the citations was then screened based on their abstracts and full text using the same criteria as for the PubMed search. This crosschecking was continued until no further articles were found from the citations of the full text articles newly found from the previous list of citations (Reference search). After 2898 citations were scrutinized, two articles remained.

Thus, 30 articles that examined the relationship between all kinds of tea intake and risk for dementia, AD, MCI, or cognitive impairment were extracted from the PubMed search and the reference search. We then narrowed the list down to articles that examined green tea intake only. Studies in which green tea drinkers had drank black and/or Chinese tea in addition to green tea were excluded.

### 2.3. Study Quality Assessment

Overall study quality was assessed according to the reporting quality and the methodological quality. The reporting quality indicates whether the necessary information for observational studies was sufficiently reported. First, we used the Strengthening the Reporting of Observational Studies in Epidemiology Statement (STROBE) checklist to score the reporting quality [9]. The reporting quality score ranges from 0 to 32 for cross-sectional studies, from 0 to 33 for case-control studies, and from 0 to 34 for cohort studies. Studies with reporting quality scores less than 13 were defined as having low overall study quality. The remaining studies were evaluated then according to the methodological quality.

The methodological quality, which determines the reliability of findings, refers to the appropriateness of the methods used in epidemiologic research. The methodological quality was qualitatively assessed based on the following aspects: sufficient temporal information between exposure and outcome, subject selection, exposure assessment, outcome diagnosis, methods for controlling confounders, and statistical analysis. Considering the importance of the temporal relationship between exposure and outcome on the causality assessment, all cross-sectional studies were considered low quality. The remaining studies that had cohort, case-cohort, nested case-control, or case-control designs were categorized into high/medium/low quality based on their methodological quality.

### 2.4. Data Extraction

The following information was tabulated for each study: author names and publication year, study settings, subject characteristics and matching parameters, green tea intake assessment, outcome diagnosis, adjusted potential confounders, reporting quality score, overall study quality, and main findings from the fully adjusted model. A meta-analysis was not conducted due to the heterogeneity among studies included and the limited number of high-quality studies. This review therefore presents only a qualitative assessment.

## 3. Results

We identified eight eligible articles [10,11,12,13,14,15,16,17]. Of these eligible studies, three were cohort studies [10,11,12] and five were cross-sectional studies [13,14,15,16,17]. The study characteristics are shown in Table 2 [10,11,12,13,14,15,16,17].

Green tea intake was assessed by self-reporting questionnaires in seven studies [10,11,12,14,15,16,17]. Lee et al., and Shen et al., also conducted interviews by trained interviewers to confirm subject self-reports [14,16]. The remaining study reported by Xu et al., did not describe the methods of assessing green tea intake [13].

The outcomes included in eight studies were AD, dementia, MCI, and/or cognitive impairment. Fischer et al., measured AD using the structured interview for the diagnosis of dementias of the Alzheimer type and multi-infarct dementia and dementias of other etiology (SIDAM) [10]. Tomata et al., Noguchi-Shinohara et al., and Lee et al., assessed dementia [11,12,14]. Tomata et al., used the long-term care insurance system of Japan to determine disabling dementia [11]. Noguchi-Shinohara et al., used the Diagnostic and Statistical Manual of Mental Disorders, 3rd edition, revised criteria for dementia [12]. Lee et al., diagnosed all-cause dementia based on the core clinical criteria recommended by the National Institute on Aging-Alzheimer’s Association workgroups [14]. Noguchi-Shinohara et al., and Xu et al., examined MCI [12,13]. Noguchi-Shinohara et al., used the general MCI criteria of the International Working Group for diagnosis of MCI [12]; however, they only analyzed the combined risk of dementia and MCI. Xu et al., assessed amnestic MCI (aMCI) according to the MCI diagnostic criteria reported by Petersen [13]. Kitamura et al., Shen et al., and Kuriyama et al., examined cognitive impairment [15,16,17]. Kitamura et al., classified participants using the Mini-Mental State Examination (MMSE) score, with values less than 24 representing cognitive impairment [15]. Shen et al., assessed cognitive impairment in their analysis of tea categories using the Chinese Cut-off of MMSE (CCM) [16]. CCM has education-specific cut-off points: persons who are illiterate with an MMSE score less than 18, persons with an education of 0 to 6 years with an MMSE score less than 21, and persons with an education of more than 6 years with an MMSE score less than 25 are categorized as having cognitive impairment [16]. Kuriyama et al., defined an MMSE score less than 26 as cognitive impairment after examining cut-off points for scores less than 24, less than 26, and less than 28 [17].

Of the three eligible cohort studies, only the study by Tomata et al., was high in overall study quality [11]. The cohort studies by Fischer et al., and by Noguchi-Shinohara et al., were considered to have a relatively medium overall study quality [10,12]. In the study by Fischer et al., the number of analyzed subjects (*n* = 2622) was only 40% of the original number (*n* = 6619) who were randomly selected from the eligible population of 138 German centers and general practitioner registered sites [10]. In addition, the subjects were a German population, most of whom do not habitually drink green tea. Noguchi-Shinohara et al., had a relatively small sample size because the potential subjects were recruited from a small area; furthermore, their participation and response rates were low [12].

All five cross-sectional studies were low in the overall study quality [13,14,15,16,17]. Xu et al., had a low reporting quality (STROBE score, 11) [13]. The overall study quality of the remaining four studies was considered low in terms of methodological quality because of insufficient information regarding the temporal relationship between exposure and outcome [14,15,16,17].

The high-quality cohort study of Tomata et al., indicated that high frequency of green tea intake was significantly associated with a low hazard ratio for dementia [11]. The two medium-quality cohort studies were inconsistent [10,12]. Fischer et al., showed that the group that drank green tea did not have a significantly different hazard ratio for AD compared to the group that did not drink green tea [10]. Noguchi-Shinohara et al., reported that the odds ratio (OR) of MCI or dementia was significantly lowered by green tea intake; however, they also reported that the OR of only dementia did not change [12]. The low-quality cross-sectional studies almost supported the positive effects of green tea. Lee et al., indicated that the group that drank green tea had a significantly lower OR for all-cause dementia than the group that did not drink green tea [14]. Kitamura et al., and Kuriyama et al., showed that an increase of green tea intake significantly lowered the OR for cognitive impairment [15,17]. The study by Xu et al., had complex results: green tea intake significantly lowered the OR for aMCI in men aged 55 to 69 years, but not in men aged 70 to 79 years, men aged ≥80 years, or the overall male population [13]. Green tea intake did not lower the OR for aMCI in women aged 55 to 69 years, 70 to 79 years, or 80 years and over, or in the overall female population [13]. Shen et al., reported that green tea intake did not affect the OR for cognitive impairment significantly [16].

## 4. Discussion

We systematically reviewed observational studies investigating the association between green tea intake and the risk for dementia, AD, MCI, or cognitive impairment in free-living populations. Six of eight eligible studies reported some kind of preventive effect of green tea intake. For this reason, it is possible that green tea intake might prevent the incidence of dementia, AD, MCI, or cognitive impairment.

Although the mechanisms of the preventive effect of green tea are not understood, four possibilities may explain these findings. The first mechanism is the antioxidant activity of green tea catechins in the brain. Oxidative stress has been demonstrated to be involved in the pathogenesis of both AD and vascular dementia (VaD) [18,19]. Green tea catechins are known as powerful free radical scavengers [19,20]. Moreover, catechins can chelate bivalent metal ions and prevent oxidation caused by reactive hydroxyl radicals [19,21]. It is poorly understood how green tea catechins are distributed in the human brain after green tea intake. Henning et al., reported that 0.02–0.56% of green tea catechins could be absorbed after green tea intake in humans [22] and Wu et al., showed that 7% of blood catechin and 11% of blood epicatechin were distributed in the rat brain after oral administration of catechin and epicatechin [23]. Regular and considerable amount of green tea intake might be important.

The second mechanism is a reduction of brain inflammation. Brain inflammation is considered to play an important role in dementia, and several inflammatory markers seem to be associated with an increased risk of all-cause dementia, especially AD [24,25]. Green tea polyphenols have anti-inflammatory effects through the inhibition of nuclear factor kappa-beta activation [19,26]. Thus, green tea intake could reduce brain inflammation.

The third mechanism is inhibition of amyloid-beta aggregation. Extracellular accumulation of amyloid plaques composed of fibrous amyloid is a pathological hallmark of AD, and amyloid oligomers and amyloid fibers generated in the process induce neurotoxicity [27]. Epigallocatechin gallate (EGCG), the main component of green tea catechins, has neuroprotective effects because EGCG inhibits amyloid-beta and aggregation [28,29].

The fourth mechanism is the maintenance of healthy blood vessels. Anti-atherosclerotic and anti-thrombotic effects are improved by green tea polyphenols [30,31]. Moreover, green tea catechins have positive effects on endothelial and overall vascular function [32,33]. These effects can prevent stroke, the main cause of VaD.

A biological relationship between green tea intake and dementia, AD, MCI, or cognitive impairment may be plausible. However, the results of eligible studies were not consistent. One of the reasons for this inconsistency might be the method by which green tea intake was estimated. Green tea intake is often estimated by frequency questionnaires, which were used in six of eight studies in the present review. Four of these six studies analyzed risk according to the frequency of green tea intake. On the other hand, the amount of tea leaves and hot water might be less standardized when making green tea compared with black tea; there is even a habit of making green tea multiple times from the same tea leaves in East Asia. For this reason, the frequency of green tea intake might not accurately reflect the intake of the constituents of green tea, some of which are considered to have positive effects against dementia, AD, MCI, and cognitive impairment. Thus, positive effects reported may not always be associated with green tea intake. Assessments of green tea intake might be more accurate if some type of marker reflecting the intake of green tea constituents could be used.

Another reason for the inconsistency between the studies might be the methods for outcome estimation. The outcomes that were included in the present review were dementia, AD, MCI, and cognitive impairment. However, the outcome of each study varied: dementia in two studies, AD in one study, MCI in one study, dementia and MCI in one study, and cognitive impairment in three studies. Moreover, the decision criteria used to define dementia and cognitive impairment varied between studies. These differences might affect the results and lead to inconsistent findings.

This systematic review has several limitations. First, our search was restricted to articles from the PubMed database. We had to identify potential articles that were not indexed in PubMed from the reference search. This limitation may have caused an omission of studies that should have been included. However, we considered that this limitation did not seriously influence the completeness of our literature search because of the thoroughness of our search.

Second, we included only studies published in English. We considered it difficult to understand non-English articles and to evaluate their quality fairly to conduct a systematic review. This limitation may have caused an omission of important non-English, high-quality studies.

Third, only one reviewer conducted the identification and evaluation of the eligible studies. Although our identification and evaluation processes were performed according to clear criteria, this limitation may have introduced a potential selection bias.

Fourth, our assessment of overall study quality might have led to an incorrect evaluation of studies. The quality assessment of observational studies is difficult because of the heterogeneity of study designs and methods. The reporting quality could be assessed quantitatively using the STROBE checklist; in contrast, the methodological quality was only estimated qualitatively. Consequently, our evaluation of the study quality might be biased even if our evaluation criteria were clearly defined. This limitation may have influenced the results and conclusions of the present review.

This systematic review has two strengths as well. To the best of our knowledge, the present review is the first systematic review that evaluates the effects of green tea intake on the risk for dementia, AD, MCI, or cognitive impairment. Reviews that evaluate all kinds of tea have already been published [4,7,34,35]; however, evaluations that do not categorize the teas used may be problematic because of the heterogeneity of tea components. We focused on green tea because it is the most common beverage in East Asia.

In addition, in conducting the reference search, we screened all citations of the full text articles not only from the PubMed search but also from the reference search. Such a search likely results in a comprehensive identification of eligible studies. A comprehensive identification is important for drawing a conclusion from a systematic literature review because all available evidence should be considered.

In conclusion, green tea intake might reduce the risk of dementia, AD, MCI, or cognitive impairment. Easily modified lifestyle habits like green tea intake may be considered to reduce the risk of these diseases. However, we could not draw a solid conclusion regarding the impact of green tea because of the limited number of eligible studies as well as the quality of the studies included. Further results from well-designed and well-conducted cohort studies are required. Ecological study between green tea intake and the incidence of AD and/or dementia worldwide might be helpful. In addition, the development of markers that measure the constituents of green tea is needed.

## Figures and Tables

**Table:** **A.** Search terms for humans, study designs, exposure, and relevant outcomes.

Number	Items	Terms
#1	Humans	Humans[mesh] OR people[tiab] OR participants[tiab] OR men[tiab] OR women[tiab] OR population[tiab] OR populations[tiab] OR individuals[tiab] OR people[ot] OR participants[ot] OR men[ot] OR women[ot] OR population[ot] OR populations[ot] OR individuals[ot]
#2	Study designs	Epidemiologic Studies[mesh] OR “case control”[tiab] OR cohort[tiab] OR cohorts[tiab] OR “cross sectional”[tiab] OR “longitudinal study”[tiab] OR “longitudinal studies”[tiab] OR “longitudinal trial”[tiab] OR “longitudinal trials”[tiab] OR “prospective study”[tiab] OR “prospective studies”[tiab] OR “prospective trial”[tiab] OR “prospective trials”[tiab] OR “retrospective study”[tiab] OR “retrospective studies”[tiab] OR “retrospective trial”[tiab] OR “retrospective trials”[tiab] OR “case control”[ot] OR cohort[ot] OR cohorts[ot] OR “cross sectional”[ot] OR “longitudinal study”[ot] OR “longitudinal studies”[ot] OR “longitudinal trial”[ot] OR “longitudinal trials”[ot] OR “prospective study”[ot] OR “prospective studies”[ot] OR “prospective trial”[ot] OR “prospective trials”[ot] OR “retrospective study”[ot] OR “retrospective studies”[ot] OR “retrospective trial”[ot] OR “retrospective trials”[ot]
#3	Exposure	tea[mesh] OR tea[tiab] OR teas[tiab] OR tea[ot] OR teas[ot]
#4	Relevant outcomes	Dementia[mesh] OR Cognition Disorders[mesh] OR dementia[tiab] OR “cognition disorder”[tiab] OR “cognition disorders”[tiab] OR “cognitive disorder”[tiab] OR “cognitive disorders”[tiab] OR “cognition impairment”[tiab] OR “cognition impairment”[tiab] OR “cognitive impairment”[tiab] OR “cognitive impairments”[tiab] OR “cognition decline”[tiab] OR “cognitive decline”[tiab] OR “cognition dysfunction”[tiab] OR “cognitive dysfunction”[tiab] OR “cognition function”[tiab] OR “cognition functions”[tiab] OR “cognitive function”[tiab] OR “cognitive functions”[tiab] OR dementia[ot] OR “cognition disorder”[ot] OR “cognition disorders”[ot] OR “cognitive disorder”[ot] OR “cognitive disorders”[ot] OR “cognition impairment”[ot] OR “cognition impairment”[ot] OR “cognitive impairment”[ot] OR “cognitive impairments”[ot] OR “cognition decline”[ot] OR “cognitive decline”[ot] OR “cognition dysfunction”[ot] OR “cognitive dysfunction”[ot] OR “cognition function”[ot] OR “cognition functions”[ot] OR “cognitive function”[ot] OR “cognitive functions”[ot]

tiab, tag for searching titles and abstracts on PubMed; ot, tag for searching other fields on PubMed.

**Table:** **B.** PubMed search strategy.

Term Combination
#1 AND #2 AND #3 AND #4

**Table:** **A.** Cohort studies.

First Author, Publication Year, [Reference No.]	Study	Subjects	Exposure Assessment	Outcome Assessment	Adjustment for Potential Confounders	Main Findings			Quality Assessment	
									STROBE Score	Study Quality
Fischer K, 2018 [10]	AgeCoDe and AgeQualiDe, German, 2003-ongoing (≥10 years follow-up).	2622 of 22,701 primary care patients living in the urban areas of the 6 German cities (Bonn, Düsseldorf, Hamburg, Leipzig, Mannheim, or Munich), aged ≥75 years.	Self-administered questionnaire of frequency at FU-1 using a short and concise 8-item “cognitive health” food intake screener.	AD according to SIDAM with consensus of the interviewing investigator and an experienced geriatrician or geriatric psychiatrist.	Age, gender, BMI, education, APOE ε4 carrier status, smoking status, physical activity score, depression, hypercholesterolemia, and a modified CCI score.	Green tea consumption	AD HR (95% CI)	*p*	21	Medium
						Non-consumption	1	-		
						Consumption	0.94 (0.86; 1.02)	0.129		
Tomata Y, 2016 [11]	Ohsaki Cohort 2006 Study, Japan, 2006–2012 (5.7 years follow-up, 67,551 person-years).	13,645 of 31,694 residents in Ohsaki City, Miyagi Prefecture, northeastern Japan, aged ≥65 years on 1 December 2006.	Self-administered FFQ (Spearman rank correlation coefficient between FFQ and food records was 0.71 for men and 0.53 for women).	Dementia defined as disabling dementia according to the criteria of LTCI system used in Japan.	Age, gender, history of disease (stroke, myocardial infarction, hypertension, diabetes, arthritis, osteoporosis, fracture), education, smoking, alcohol drinking, BMI, psychological distress score, time spent walking, social support, participation in community activities, motor function score, consumption volume of specific foods (green and yellow vegetables and fruit), coffee consumption, and energy intake.	Green tea consumption	Dementia HR (95% CI)	*p* for trend	18	High
						<1 cup/day	1	<0.001		
						1–2 cups/day	1.06 (0.89–1.27)			
						3–4 cups/day	0.88 (0.74–1.04)			
						≥5 cups/day	0.73 (0.61–0.87)			
Noguchi-Shinohara M, 2014 [12]	Nakajima Project, Japan, 2007–2013 (mean 4.9 (0.9) years follow-up).	723 of 2845 residents of Nakajima, aged ≥60, completed cognitive tests, without dementia, MCI, or MMSE score <24.	Self-administered questionnaire of frequency, reviewed by trained researchers.	Dementia: DSM-III-R MCI: general MCI criteria of International Working Group.	Age, gender, history of hypertension/diabetes mellitus/hyperlipidemia, education, ApoE, alcohol drinking, smoking, physical activities/hobbies, and coffee/black tea/green tea consumption.	Green tea consumption	Dementia OR (95% CI)	*p*	21	Medium
						None	1	-		
						1–6 d/w	0.90 (0.34, 2.35)	0.824		
						Every day	0.26 (0.06, 1.06)	0.06		
						Green tea consumption	MCI or dementia OR (95% CI)	*p*		
						None	1	-		
						1–6 d/w	0.47 (0.25, 0.86)	0.015		
						Every day	0.32 (0.16, 0.64)	0.001		

**Table:** **B.** Cross-sectional studies.

First Author, Publication Year, [Reference No.]	Study	Subjects	Exposure Assessment	Outcome Assessment	Adjustment for Potential Confounders	Main Findings			Quality Assessment	
									STROBE Score	Study Quality
Xu H, 2018 [13]	CLAS, China, 2011–2012.	1003 of randomly selected 4411 residents from 20 target communities in the eastern, mid, and western parts of China, aged ≥60.	Unclear (reviewed type of tea consumed with frequency of tea consumption).	aMCI diagnostic criteria reported by Petersen with MMSE, MoCA, ADL, GDS, HIS, and MRI scans.	Education.	Green tea consumption	MCI OR (95% CI)	*p*	11	Low
						All male				
						Non-consumption	1	-		
						Consumption	0.657 (0.46–0.93)	0.019		
						55–69 years male				
						Non-consumption	1	-		
						Consumption	0.376 (0.20–0.70)	0.002		
						70–79 years male				
						Non-consumption	1	-		
						Consumption	0.802 (0.64–1.79)	0.802		
						≥80 years male				
						Non-consumption	1	-		
						Consumption	0.652 (0.28–1.51)	0.318		
						All female				
						Non-consumption	1	-		
						Consumption	0.82 (0.58–1.16)	0.261		
						55–69 years female				
						Non-consumption	1	-		
						Consumption	1.06 (0.62–1.80)	0.840		
						70–79 years female				
						Non-consumption	1	-		
						Consumption	0.96 (0.56–1.65)	0.890		
						≥80 years female				
						Non-consumption	1	-		
						Consumption	0.43 (0.18–1.03)	0.057		
Lee CY, 2017 [14]	A nationwide, population-based, door-to-door, in-person survey in Taiwan, 2011–2013.	7964 of 28,600 residents across Taiwan, aged ≥65.	Interview using a structured questionnaire, conducted by well-trained field interviewers according to an operational manual.	All-cause dementia: the core clinical criteria recommended by NIA-AA.	Age, gender, education, BMI, dietary habits, habitual exercises, and co-morbidities, including hypertension, diabetes, and cerebrovascular diseases.	Green tea consumption	All-cause dementia OR (95% CI)	*p*	18	Low
						Non-consumption	1	-		
						Consumption	0.51 (0.34-0.75)	0.00		
Kitamura K, 2016 [15]	PROST, Japan, 2008–2014.	1143 of 2161 patient registry of Sado General Hospital, aged ≥40, not undergoing kidney dialysis.	Self-administered questionnaire of frequency.	Cognitive impairment: MMSE score <24 (MMSE cutoff score of 23/24).	Age, BMI, history of stroke and myocardial infarction, walking time, alcohol, and fruit consumption.	Green tea consumption	Cognitive impairment OR (95% CI)	*p*	19	Low
						0 = none, 1 = 1–6 times/wk, 2 = 7 times/wk	0.83 (0.70–0.98)	0.032		
Shen W, 2015 [16]	ZPHS, China, 2014.	9375 of randomly selected 1500 residents from each of 7 sites in Zhejian province, aged ≥60.	Self-reported frequency/type/volume/preferred concentration in interview by trained researchers.	Cognitive impairment (CCM): MMSE score <18 for illiteracy, <21 for 0–6 years educated, <25 for >6 year educated Cognitive impairment (worldwide): MMSE score <24.	Age, gender, ethnicity, education, marital status, BMI, WHR, SBP, DBP, income, having children, diabetes/CHD/AD/PD, family diabetes/CHD/AD/PD history, smoking, alcohol drinking, activity, vegetable intake, fruit intake, red meat intake, bean intake, milk intake, supplement use, depression, ADL (all analyses), tea types, tea concentration (Tea consumption volume), tea consumption volume, tea concentration (Tea types), tea consumption volume, and tea types (Tea concentration).	Tea types	Cognitive impairment (CCM) OR (95% CI)	*p*	19	Low
						Non-consumption	1	-		
						Green tea	1.04 (0.72, 1.51)	Not shown		
Kuriyama S, 2006 [17]	Tsurugaya Project, Japan, 2002.	1103 of 2730 residents of Tsurugaya, aged ≥70, with information on tea consumption, cognitive function, body weight, height, blood glucose, blood pressure, depressive symptoms.	Self-administered semi-quantitative questionnaire.	Cognitive impairment: MMSE score <26.	Age, gender, green tea/black or oolong tea consumption, coffee consumption, diabetes mellitus, hypertension, history of stroke, depressive symptoms, education, visiting friends, energy intake, VC/VE supplementation, and fish intake.	Green tea consumption	Cognitive impairment OR (95% CI)	*p* for trend	19	Low
						≤3 cups/w	1	0.0006		
						4–6 cups/w or 1 cup/d	0.62 (0.33, 1.19)			
						≥2 cups/d	0.46 (0.30, 0.72)			

STROBE, strengthening the reporting of observational studies in epidemiology statement; AgeCoDe, study on ageing, cognition and dementia in primary care patients; AgeQualiDe, study on needs, health service use, costs, and health-related quality of life in a large sample of oldest-old primary care patients; FU-1, follow-up-1; AD, Alzheimer’s disease; SIDAM, structured interview for the diagnosis of dementias of the Alzheimer type and multi-infarct dementia and dementias of other etiology; BMI, body mass index; APOE ε4, apolipoprotein E ε4; CCI, Charlson comorbidity index; HR, hazard ratio; CI, confidence interval; FFQ, food frequency questionnaire; LTCI, long-term care insurance; MCI, mild cognitive impairment; MMSE, Mini-Mental State Examination; DSM-III-R, Diagnostic and Statistical Manual of Mental Disorders 3rd edition, revised; OR, odds ratio; CLAS, China longitudinal aging study; MoCA, Montreal cognitive assessment; ADL, activities of daily living scale; GDS, global deterioration scale; HIS, Hachinski ischemia scale; MRI, magnetic resonance imaging; aMCI, amnestic mild cognitive impairment; NIA-AA, National Institute on Aging-Alzheimer’s Association workgroups; ZPHS, Zhejiang Major Public Health Surveillance Program; CCM, Chinese cut-off of MMSE; WHR, waist-to-hip ratio; SBP, systolic blood pressure; DBP, diastolic blood pressure; CHD, coronary heart disease; PD, Parkinson’s disease; VC, vitamin C; VD, vitamin D.

## References

[1] World Health Organization Dementia, Fact Sheet on Dementia. https://www.who.int/news-room/fact-sheets/detail/dementia.

[2] Santos C., Costa J., Santos J., Vaz-Carneiro A., Lunet N. (2010). Caffeine intake and dementia: Systematic review and meta-analysis. J. Alzheimers Dis..

[3] Kim Y.-S., Kwak S.M., Myung S.-K. (2015). Caffeine intake from coffee or tea and cognitive disorders: A meta-analysis of observational studies. Neuroepidemiology.

[4] Panza F., Solfrizzi V., Barulli M.R., Bonfiglio C., Guerra V., Osella A., Seripa D., Sabbà C., Pilotto A., Logroscino G. (2015). Coffee, tea, and caffeine consumption and prevention of late-life cognitive decline and dementia: A systematic review. J. Nutr. Health Aging.

[5] Braidy N., Jugder B.E., Poljak A., Jayasena T., Mansour H., Nabavi S.M., Sachdev P., Grant R. (2016). Resveratrol as a Potential Therapeutic Candidate for the Treatment and Management of Alzheimer’s Disease. Curr. Top. Med. Chem..

[6] Mandel S., Youdim M.B. (2004). Catechin polyphenols: Neurodegeneration and neuroprotection in neurodegenerative diseases. Free Radic. Biol. Med..

[7] Mancini E., Beglinger C., Drewe J., Zanchi D., Lang U.E., Borgwardt S. (2017). Green tea effects on cognition, mood and human brain function: A systematic review. Phytomedicine.

[8] National Institutes of Health’s National Library of Medicine PubMed. https://www.ncbi.nlm.nih.gov/pubmed/.

[9] von Elm E., Altman D.G., Egger M., Pocock S.J., Gøtzsche P.C., Vandenbroucke J.P., STROBE Initiative (2007). Strengthening the Reporting of Observational Studies in Epidemiology (STROBE) statement: Guidelines for reporting observational studies. BMJ.

[10] Fischer K., Melo van Lent D., Wolfsgruber S., Weinhold L., Kleineidam L., Bickel H., Scherer M., Eisele M., van den Bussche H., Wiese B. (2018). Prospective Associations between Single Foods, Alzheimer’s Dementia and Memory Decline in the Elderly. Nutrients.

[11] Tomata Y., Sugiyama K., Kaiho Y., Honkura K., Watanabe T., Zhang S., Sugawara Y., Tsuji I. (2016). Green Tea Consumption and the Risk of Incident Dementia in Elderly Japanese: The Ohsaki Cohort 2006 Study. Am. J. Geriatr. Psychiatry.

[12] Noguchi-Shinohara M., Yuki S., Dohmoto C., Ikeda Y., Samuraki M., Iwasa K., Yokogawa M., Asai K., Komai K., Nakamura H. (2014). Consumption of green tea, but not black tea or coffee, is associated with reduced risk of cognitive decline. PLoS ONE.

[13] Xu H., Wang Y., Yuan Y., Zhang X., Zuo X., Cui L., Liu Y., Chen W., Su N., Wang H. (2018). Gender differences in the protective effects of green tea against amnestic mild cognitive impairment in the elderly Han population. Neuropsychiatr. Dis. Treat..

[14] Lee C.-Y., Sun Y., Lee H.-J., Chen T.-F., Wang P.-N., Lin K.-N., Tang L.-Y., Lin C.-C., Chiu M.-J. (2017). Modest Overweight and Healthy Dietary Habits Reduce Risk of Dementia: A Nationwide Survey in Taiwan. J. Prev. Alzheimers Dis..

[15] Kitamura K., Watanabe Y., Nakamura K., Sanpei K., Wakasugi M., Yokoseki A., Onodera O., Ikeuchi T., Kuwano R., Momotsu T. (2016). Modifiable Factors Associated with Cognitive Impairment in 1,143 Japanese Outpatients: The Project in Sado for Total Health (PROST). Dement Geriatr. Cognit. Disord. Extra.

[16] Shen W., Xiao Y., Ying X., Li S., Zhai Y., Shang X., Li F., Wang X., He F., Lin J. (2015). Tea Consumption and Cognitive Impairment: A Cross-Sectional Study among Chinese Elderly. PLoS ONE.

[17] Kuriyama S., Hozawa A., Ohmori K., Shimazu T., Matsui T., Ebihara S., Awata S., Nagatomi R., Arai H., Tsuji I. (2006). Green tea consumption and cognitive function: A cross-sectional study from the Tsurugaya Project 1. Am. J. Clin. Nutr..

[18] Luca M., Luca A., Calandra C. (2015). The Role of Oxidative Damage in the Pathogenesis and Progression of Alzheimer’s Disease and Vascular Dementia. Oxid. Med. Cell. Longev..

[19] Molino S., Dossena M., Buonocore D., Ferrari F., Venturini L., Ricevuti G., Verri M. (2016). Polyphenols in dementia: From molecular basis to clinical trials. Life Sci..

[20] Nanjo F., Mori M., Goto K., Hara Y. (1999). Radical scavenging activity of tea catechins and their related compounds. Biosci. Biotechnol. Biochem..

[21] Kumamoto M., Sonda T., Nagayama K., Tabata M. (2001). Effects of pH and metal ions on antioxidative activities of catechins. Biosci. Biotechnol. Biochem..

[22] Henning S.M., Niu Y., Lee N.H., Thames G.D., Minutti R.R., Wang H., Go V.L., Heber D. (2004). Bioavailability and antioxidant activity of tea flavanols after consumption of green tea, black tea, or a green tea extract supplement. Am. J. Clin. Nutr..

[23] Wu L., Zhang Q.L., Zhang X.Y., Lv C., Li J., Yuan Y., Yin F.X. (2012). Pharmacokinetics and blood-brain barrier penetration of (+)-catechin and (−)-epicatechin in rats by microdialysis sampling coupled to high-performance liquid chromatography with chemiluminescence detection. J. Agric. Food Chem..

[24] Koyama A., O’Brien J., Weuve J., Blacker D., Metti A.L., Yaffe K. (2013). The role of peripheral inflammatory markers in dementia and Alzheimer’s disease: A meta-analysis. J. Gerontol. A Biol. Sci. Med. Sci..

[25] Darweesh S.K.L., Wolters F.J., Ikram M.A., de Wolf F., Bos D., Hofman A. (2018). Inflammatory markers and the risk of dementia and Alzheimer’s disease: A meta-analysis. Alzheimers Dement..

[26] Spagnuolo C., Moccia S., Russo G.L. (2018). Anti-inflammatory effects of flavonoids in neurodegenerative disorders. Eur. J. Med. Chem..

[27] Klein W.L., Stine W.B., Teplow D.B. (2004). Small assemblies of unmodified amyloid beta-protein are the proximate neurotoxin in Alzheimer’s disease. Neurobiol. Aging.

[28] Afzal M., Safer A.M., Menon M. (2015). Green tea polyphenols and their potential role in health and disease. Inflammopharmacology.

[29] Ehrnhoefer D.E., Bieschke J., Boeddrich A., Herbst M., Masino L., Lurz R., Engemann S., Pastore A., Wanker E.E. (2008). EGCG redirects amyloidogenic polypeptides into unstructured, off-pathway oligomers. Nat. Struct. Mol. Biol..

[30] Stangl V., Lorenz M., Stangl K. (2006). The role of tea and tea flavonoids in cardiovascular health. Mol. Nutr. Food Res..

[31] Fraser M.L., Mok G.S., Lee A.H. (2007). Green tea and stroke prevention: Emerging evidence. Complement. Ther. Med..

[32] Moore R.J., Jackson K.G., Minihane A.M. (2009). Green tea (*Camellia sinensis*) catechins and vascular function. Br. J. Nutr..

[33] Bielli A., Scioli M.G., Mazzaglia D., Doldo E., Orlandi A. (2015). Antioxidants and vascular health. Life Sci..

[34] Song J., Xu H., Liu F., Feng L. (2012). Tea and cognitive health in late life: Current evidence and future directions. J. Nutr. Health Aging.

[35] Polito C.A., Cai Z.-Y., Shi Y.-L., Li X.-M., Yang R., Shi M., Li Q.-S., Ma S.-C., Xiang L.-P., Wang K.-R. (2018). Association of Tea Consumption with Risk of Alzheimer’s Disease and Anti-Beta-Amyloid Effects of Tea. Nutrients.

